# P159: The views of senior hospital managers on innovative strategies to improve hand hygiene adherence: a qualitative study

**DOI:** 10.1186/2047-2994-2-S1-P159

**Published:** 2013-06-20

**Authors:** E McInnes, R Phillips

**Affiliations:** 1 School of Nursing, Midwifery and Paramedicine, Australian Catholic University, Sydney, Australia; 2 Nursing Research Institute SVMHS & ACU, Darlinghurst, Australia

## Introduction

Hand hygiene (HH) adherence remains low among hospital clinicians and improvement strategies such as audit and education have weak or mixed effects.^1^ Innovative approaches are required to improve HH and reduce hospital-acquired infection rates; hence we sought the views of senior hospital managers about new ways to improve HH.

## Objectives

To identify the views of senior hospital managers on: 1) the concept of HH non-adherence as a healthcare error; and 2) innovative strategies to include within HH improvement programs.

## Methods

We conducted a qualitative study at a tertiary referral hospital. Twelve purposively sampled senior clinical and executive staff participated in semi-structured interviews that were audio-recorded and transcribed. Data were thematically analysed.

## Results

Four themes emerged. *Making hand hygiene part of the mantra* reflects perceptions that HH culture and practice is variable across disciplines and within different parts of the facility. *Shifting the balance of responsibility* reflects views that introducing the concept of HH non-adherence as a healthcare error would strengthen HH programs. Innovative approaches suggested were: 1) *Overdue to start using the hammer:* refers to a tiered system of disciplinary action which may include financial fines and suspension from practice for repeated lapses, combined with HH education as a mandatory part of clinical reaccreditation; and 2) *Role modelling and empowering all hospital staff* through assertiveness training to remind and prompt each other about HH. This would be supported and sanctioned by hospital policies.

## Conclusion

Basing HH strategies on the concepts of individual responsibility and non-adherence as a healthcare error was perceived by senior hospital leaders as necessary to reinvigorate and increase the impact of current HH programs. Future developments will involve evaluating the feasibility of these approaches within HH improvement programs.

## Disclosure of interest

None declared
